# Polymer/Graphene oxide nanocomposite thin film for NO_2_ sensor: An in situ investigation of electronic, morphological, structural, and spectroscopic properties

**DOI:** 10.1038/s41598-020-59726-5

**Published:** 2020-02-19

**Authors:** Praveen Kumar Sahu, Rajiv K. Pandey, R. Dwivedi, V. N. Mishra, R. Prakash

**Affiliations:** 1 Department of Electronics Engineering, Indian Institute of Technology (Banaras Hindu University), Varanasi, 221005 India; 2 School of Materials Science and Technology, Indian Institute of Technology (Banaras Hindu University), Varanasi, 221005 India

**Keywords:** Environmental sciences, Materials science, Nanoscience and technology

## Abstract

The higher operating temperature of metal oxide and air instability of organic based NO_2_ sensor causes extremely urgent for development of a reliable low cost sensor to detect NO_2_ at room temperature. Therefore, we present a fabrication of large area Polymer/GO nano hybrid thin film for polymer thin film transistors (PTFTs) based NO_2_ sensors assisted via facile method named ‘spreading-solidifying (SS) method’, grown over air/liquid interface and successive investigation of effect of NO_2_ on film via several characterizations. The PTFTs sensor has demonstrated swift and high response towards low concentration of NO_2_ gas with air stability and provided real time non-invasive type NO_2_ sensor. Herein, we are reporting the nanohybrid PBTTT/GO composite based PTFT sensor with good repeatability and sensor response for low concentration NO_2_. The thin film grown via SS technique has reported very good adsorption/desorption of target analyte having response/recovery time of 75 s/523 s for 10 ppm concentration of NO_2_ gas. It has been observed that % change in drain current (sensor response) saturated with increasing concentration of NO_2_. The transient analysis demonstrates the fast sensor response and recovery time. Furthermore, in order to understand the insight of high performance of sensor, effect of NO_2_ on nanohybrid film and sensing mechanism, an *in situ* investigations was conducted via multiple technique viz. spectral, electronic, structural, and morphological characterization. Finally, the performance of sensor and the site of adsorption of NO_2_ at polymer chains were argued using schematic diagram. This work shows the simple fabrication process for mass production, low cost and room temperature operated gas sensors for monitoring the real-time environment conditions and gives an insight about the sensing mechanism adsorption site of NO_2_.

## Introduction

NO_x_ is known as hazardous gas, mostly emanating from the improper combustion of the fossil fuels, decomposition of explosive and cause acid rain after reaction with moisture which is very harmful to human life and ecosystems^1–5^. The detection of low concentration of NO_2_ gas is extremely important to avoid long term effect of human health. The maximum permissible limit set by the US administration for the average 8 hours exposure with NO_2_ gas is 5 ppm^6^. Metal oxide transistors are generally operated at higher temperature and integration with other planar devices is found cumbersome. It also suffers from poor selectivity as well as high power consumption. The chemiresistive based metal oxide sensors are widely investigated and adapted by the industries due to high sensitivity, easy production and long service life. Meanwhile, the higher operating temperature (>300 °C) increases the cost and has limitation to work in low temperature or flammable environment. Considering these challenges, organic field effect transistors based sensors have been reported as potential replacement but poor repeatability and sluggish response limit their applications. Therefore, there is a requirement to develop highly sensitive and room temperature operated NO_x_ gas sensor for civil and environmental applications. In order to sort out the above mentioned problem, solution processable conducting polymer also endeavours in this direction but air instability, requirement of passivation layer and recovery again need additional processing cost and restricted their application. This problem may remove nicely by compositing the materials with 2D nanomaterials without need of additional cost with enhancement in air stability. In this regard, graphene oxide (GO) nanosheet has emerged as one of the best two-dimensional nano-materials having sp^2^ conjugated domains and scattered polar oxygenated domains along with large surface to volume ratio. The inherent larger surface area with nm level height profile of GO plays significant role in enhancing the various properties of the polymer matrix such as increase the thermal and electrical conductivity and the dimensional stability of the composite when compared to the polymer matrix^7,8^. The well-known exceptional functional properties and the excellent structure of selected 2D- nanomaterial bestow charge transfer interactions with various conducting polymers^9,10^. Further, it is worth mentioning that the band gap of the polymer and work function of GO falls within the range endows successful charge transfer (CT) complexes interaction.

Recently, among solution processable organic conjugated molecules^11–17^ namely poly[2,5-bis(3-tetradecylthiophen-2-yl)thieno[3,2-b]thiophene] (PBTTT) has evolved as promising conducting polymer having immense scientific interest^13,18–21^. It has added advantage of formation of self-organized, ordered structures with superior charge transfer characteristics via π-π stacking and presence of alkyl side chains^22–24^. Now, in order to harvest the unique characteristics of two-dimensional GO nanosheets and promising conjugated polymer PBTTT, we have directed towards fabricating of thin film of hybrid nano-composite by selecting these two materials for PTFTs as NO_2_ gas sensor. It is worth full to mention here that the practical implementation of these nanohybrid materials in large area, flexible electronic devices and their applications is directly associated with the capability of producing materials at a cost which is significantly below with respect to traditional electronic devices/sensors design. Contemporary, the existing challenges being faced during fabricating the thin film via several techniques like bar-coating^25^, spin coating^26–28^, drop casting^12,29–31^, LB technique^32–34^ etc., we have introduced a very facile thin film processing methodology for solution processable conjugated composite polymer matrices. Several researches for NO_2_ gas detection is carried out and the authors are come up with final conclusion of film thickness also govern sensitivity with the target analyte. Xie *et al*.^35^ have reported the high response for thinner film. However, Yang *et al*.^36^ have reported the nano hybrid for enhanced sensing response towards NO_2_ gas. The analyte interactions with the composite polymer thin film is directly associated with the thickness of the active matrix. In PTFT based device geometry, the charge transport layer is confined between insulating gate dielectric and composite polymer semiconductor. In this regard spreading and solidifying method is potential candidates for high performance PTFTs based NO_2_ sensor. It offers self-generation and spontaneous formation of thin films by the accretion of targeted materials, ease arrangement with negligible materials wastage. It also offers the choices to control the grown thin film thickness by optimizing the number of coatings thereof. The detail procedures adopted for the above described technique have been discussed broadly in results and discussion section.

In our work, we have fabricated the polymer thin film transistor assisted via SS method using PBTTT/GO nanohybrid and is further used as NO_2_ sensor. The sensor has demonstrated swift and high response towards NO_2_ gas with air stability and provided real time non-invasive type NO_2_ sensor. Furthermore, an analytical approach to understand the sensing mechanism of NO_2_, an *in situ* investigation was conducted via multiple technique viz. spectral characterization using electronic absorption spectra, FT-IR, Raman, electronic characterization using Cyclic Voltammetry, structural characterization using GIXD and morphological characterization using AFM, Phase imaging and KPFM and Phase contrast which reveals the insight of high performance and site of adsorption of NO_2_ at polymer chains.

## Results

### Nucleation and growth phenomena of composite thin film

**Figure 1:**
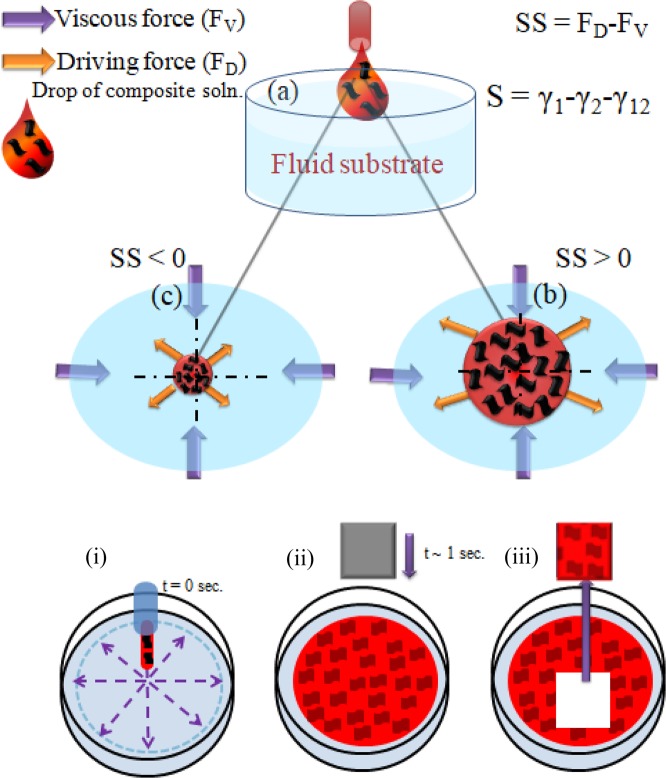
(**a**) Illustration of nucleation and growth mechanism of PBTTT/GO nanohybrid thin film over mobile liquid substrate. Nucleation and growth phenomena is governed by equation S = γ_1_-γ_2_-γ_12_ where γ_1_, γ_2_ are surface tension of base liquid substrate and solution, and γ_12_ is surface tension at interface of liquid substrate and solution. (**b**) Positive S and optimized evaporation rate of solution results in the formation of uniform composite polymer thin film. However, the negative value of S results (**c**) coagulated structure. Schematics for whole procedure of SS method (i–iii).

According to Marangoni flow, the solvent (like hydrophobic dissolvable) having low surface energy is spontaneously spreaded over the higher surface energy solvents like deionized ultrapure water (18 Mohm cm)^37–39^. The surface pressure gradient is created while dropping of low energy solvent over high energy solvent at the interface of surrounding materials. The developed surface pressure gradient results the spontaneous spreading of low energy solvent towards the highly strained surface^39,40^. Further, the overextension of the solvent is restricted by viscous force (F_V_) contemporarily^41,42^. The two driving and viscous forces (F_D_ and F_V_), acts against to each other during spontaneous spreading (SS); and governed by the equation SS = F_D_ − F_V_. Moreover, the spreading coefficient (S) depends upon the driving force F_D_ as governed by the equation S = γ_1_-γ_2_-γ_12_, where γ_1_ signifies the surface tension of the liquid (DI water) having surface tension of 72.2 ± 0.3 dyn/cm at 23 °C acting outward, γ_2_ signifies the surface tension of hybrid nanocomposite polymeric material dissolved in chloroform acting inward and γ_12_ signifies the surface tension developed at the interface boundaries of the liquids of different surface free energy while dropping the droplets of hydrophobic polymeric solution over hydrophilic aqueous substrate; which is illustrated in Fig. 1. Here, the viscous force, F_V,_ solely depends upon the viscosity of base aqueous substrate along with γ_2_ and γ_12_ directs the path for hydrophobic hybrid nanocomposite polymeric solution over the base aqueous substrate. The process involved to achieve air-processable, self-assembled, large area composite nanohybrid thin film is only possible due to ultrafast evaporation and simultaneous solidification of polymeric composite solution. For spreading of the hydrophobic polymeric composite solution, the spontaneous spreading (SS) must be greater than zero (SS > 0) as illustrated in Fig. 1(b). The hybrid nanocomposite polymeric solution will not spread if SS is less than zero, which is shown in Fig. 1(c). By optimizing the polymeric nanohybrid solvent as well as aqueous (water based) substrate, a compact high quality self-assembled polymer thin film has been fabricated. The whole procedure of spreading and solidifying technique is summarized in Fig. 1(i–iii).

### NO_2_ sensing property investigation of PBTTT/GO nanohybrid thin film

In order to investigate the NO_2_ sensing property of PBTTT/GO nanohybrid thin film, PTFTs were fabricated with interdigitated source drain electrode. Preliminary investigation by I_d_-V_d_ and I_d_-V_g_ reveals the p type semiconducting behavior of nanohybrid thin film (Fig. S3), having on/off ratio of ~10^3^ in ambient condition. The PTFTs device parameters were extracted in saturation region of operation at different concentration of gas. Parameter like threshold voltage (*V*_*th*_) has been obtained by using following equations as^43^:

1 $${I}_{DS}^{sat.}=\frac{{\mu }_{sat.}{C}_{i}W}{2L}{({V}_{GS}-{V}_{th})}^{2};\,{V}_{DS}\ge ({V}_{GS}-{V}_{th})$$

where, *W* and *L* represent the channel width and length of fabricated PTFT sensors, respectively.

2 $$\sqrt{{I}_{DS}}=\sqrt{\frac{W}{2L}{C}_{i}\mu }({V}_{GS}-{V}_{th})$$

The threshold voltage (*V*_*th*_) is obtained using the above Eqs. (1) & (2).

**Figure 2:**
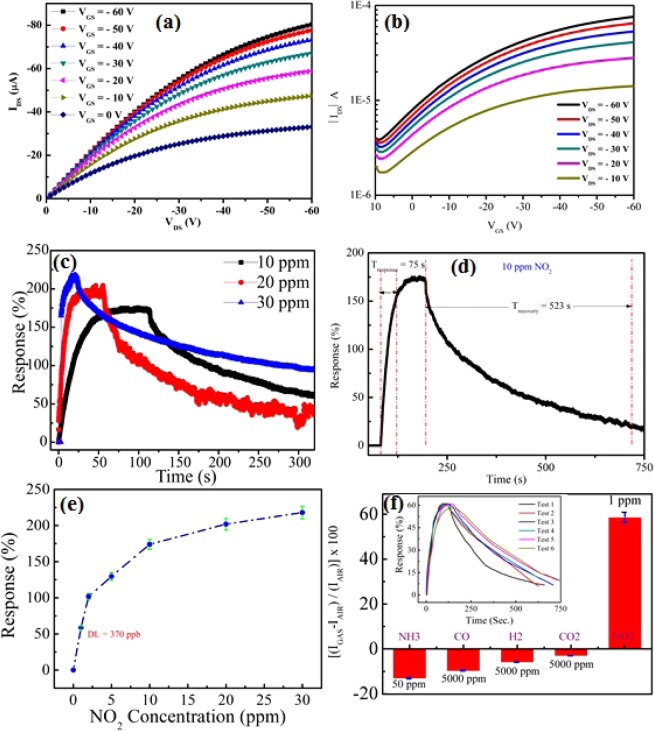
(**a**) I_DS_-V_DS_ & **(b)** I_DS_-V_GS_ curve of PTFTs sensor after exposure with 30 ppm concentration, **(c)** Transient response after exposure with different concentration, **(d)** Transient analysis after exposure with 10 ppm concentration of NO_2_ gas, **(e)** Response curve for 1–30 ppm concentration of NO_2_ gas, and **(f)** Selectivity test of the sensor among various interfering gases. Inset shows the repeatability test of the sensor for six test cycles with 1 ppm concentration of NO_2_ gas.

Here, we reported a highly selective PTFT sensor based on polymer/GO crystalline nanohybrid thin film for NO_2_ (a common air pollutant and oxidizing gas) detection. The change in threshold voltage of PTFT sensor is observed and found the positive shift ranging from −1 V to +23 V after exposer with 30 ppm concentration of NO_2_ gas. The sensor has shown saturated response characteristics for every trial during sensing measurement which may be possible due to adsorption of NO_2_ gas on the nanohybrid thin film. It is noteworthy that NO_2_ is electron withdrawing agent and generates holes over nanohybrid film after interaction. We examined the NO_2_ sensing behavior of PTFTs sensor which demonstrated remarkable sensing property with prompt and significant enhancement in drain current (I_d_) after the introduction of trace amount of target NO_2_ gas at room temperature. The detailed PTFTs sensing characteristics with the exposure to different concentration (1–30 ppm) of NO_2_ gas are appeared in Figs. 2 and S4.

#### Quantitative analysis of hybrid polymer/graphene oxide NO_2_ sensor

To analyze the sensing performance of fabricated PTFT sensors, we have calculated the gas response which is extracted from the Eq. 3. Here, the output characteristic (I_d_–V_d_) of PTFTs in common source configuration were employed to probe the response of the fabricated sensors for NO_2_ gas. The response of fabricated PTFTs sensors towards NO_2_ gas are extracted by applying the equation as^44^.

3 $$S=\frac{|{I}_{N{O}_{2}}-{I}_{Air}|}{|{I}_{Air}|}\times 100 \% $$

where, ${I}_{Air}$ and ${I}_{N{O}_{2}}$ are the measured drain current in the ambient and after the exposure with NO_2_ gas, respectively.

The sensor exhibits very high response of 58.54%, 101.72%, 129.49%, 174%, 202% and 218% for 1 ppm, 2 ppm, 5 ppm, 10 ppm, 20 ppm and 30 ppm exposure of NO_2_ gas, respectively. Further, a response curve has been plotted for the concentration ranging from 1ppm to 30 ppm of NO_2_ gas and is illustrated in Fig. 2(e). The key parameters of any sensors design is limit of detection (LOD), and it is very useful to detect lower concentration of gas to avoid health hazards for long term exposure. The LOD value is calculated after obtaining the root-mean-square-deviation by extracting the noise level of baseline and by fitting the sensitivity curve while the signal to noise ratio becomes 3. The sensor shows LOD of 0.37 ppm. However, the maximum permissible limit set by the US administration for the average 8 hours exposure with NO_2_ gas is 5 ppm. With the exposure of higher concentration of NO_2_ gas (>10 ppm), the sensor responses are not linear as it can be seen in response curve in contrary with lower concentration. The effect of step addition of 10 ppm concentration in the sensing chamber to the as fabricated NO_2_ sensor has been plotted, which is shown in Fig. S4.

It is noteworthy to mention that PTFTs sensor shows the swift response for increased concentration of NO_2_ gas; however recovery time delays was observed in subsequent increase in target gas concentration as shown in Fig. 2(c). The introduction of GO flakes in PBTTT polymer matrices increased the effective surface-to-volume ratio as it is highly desirable in gas sensing application area. Here, it should be noted that both the chosen materials are highly sensitive to NO_2_ gas. The NO_2_ molecules are engaged with interruption of polymer chain morphology as well as absorption toward GO flakes. The electron withdrawing characteristics of NO_2_ gas is responsible for swift response towards the fabricated PTFTs sensor. It is seen that the concentration of mobile charge carriers (holes) gets populated with increase in target gas concentration. The increased concentration of holes inside the hybrid PBTTT/GO thin film is not permanent as we flush the sensor with fresh air. However, it also shows baseline drift of sensor response. In this way, it is confirmed that the some non-reversible reactions might take place.

#### The performance analysis of hybrid polymer/graphene oxide NO_2_ sensor

The sensor’s response and recovery time plays critical role in gas sensing. The transient analysis and response of sensors was carried out by considering the drain current after exposing with different concentration of NO_2_ gas at fixed test parameter such as constant gate voltage (V_gs_ = −60 V) and drain voltage (V_ds_ = −60 V) supply. Various organic semiconductor based sensor operated at room temperature offer larger response and recovery time for more than half an hour. The response time (t_res._) is defined on the basis of time required to achieve the 90% of final saturated value of current, whereas the recovery time (t_rec._) is defined as the time required by decreasing the saturated value of current to 10%. Our sensor shows the t_res._ and t_rec._ for 10 ppm of NO_2_ gas exposure is 75 s and 523 s, respectively. A comparative sensing performance of various NO_2_ sensor based on state-of-the-art nanocomposite/layered materials is summarized in Table 1 below.

**Table 1:** Comparison with previously reported results.

Sensor type	Materials	Response	Response time	Recovery time	Method	Remarks [reference]
OFET	RGO/P3HT bilayer	~100% (20 ppm)	60 min	90 min	Airbrush	Poor response & recovery^46^
OFET	P3HT-ZnO@GO	210% (5 ppm)	5 min	—	Spin coat	Incomplete recovery^36^
OFET	ZnO NS-NR/P3HT	180% (50 ppm)	15 min	—	Spin coat	Incomplete recovery^47^
MSM	VOPc/PTCDI-Ph	670% (30 ppm)	~20 min	~50 min	Vacuum Evaporation	Complex fabrication^48^
MSM	SnO_2_/ZnO	238.7% (1 ppm)	100 s	220 s	Thermal evaporation/ALD	Response under UV^49^
MSM	P3HT:ZnO-nanowire	~32% (4 ppm)	~1.2 min	~7 min	Drop casting	Low sensitivity^50^
MSM	Polythiophene (PTh)	9% (10 ppm)	297 s	1603 s	Spin coat	Low sensitivity^51^
MSM	p-Type SnS_2_/rGO	47% (1 ppm)	149 s	—	Drop casting	Low sensitivity^52^
Our report	PBTTT/GO	174% (10 ppm)	75 s	523 s	SS method	High performance

Repeatability and selectivity are two crucial parameters for designing a reliable gas sensor. A back-to-back test of 1 ppm concentration of NO_2_ gas for 6 cycles has been carried out to check the repeatability of the fabricated sensor and it is found that sensor has shown average response of 58.43%. After analyzing the sensor response for all the test cycles, the sensor response falls in the range of ±4% of the average value. The sensor has shown good repeatability characteristics for 1 ppm of target gas which can be seen in inset of Fig. 2(f). Meanwhile, the sensor has shown more base line drift when it is exposed with higher concentration of target gas. This may be arising due to residual gas molecules present to the exposed film and get weaken due to prolonged exposure with target gas and desorption time.

The selectivity test of the proposed sensor has also been incorporated by considering the various interfering gases (environment pollutants, toxic and explosive gases) like NH_3_, CO, H_2_, and CO_2_. The sensor has shown excellent selectivity towards these reducing gases. The sensor response for 1 ppm NO_2_ gas along with 50 ppm NH_3_, 5000 ppm of other reducing gases like CO, H_2_, and CO_2_ is illustrated in Fig. 2(f). The adsorption of NO_2_ molecules in hybrid PBTTT/GO film leads to increase in drain current by increasing the hole carrier concentration in the composite film. The converse is pertinent for other reducing gases, like, NH_3_, where it acts as reducing gas by increasing the electron concentration.

### Investigation of effect of NO_2_ on nano hybrid SS Film

#### Electronic absorption spectrum

**Figure 3:**
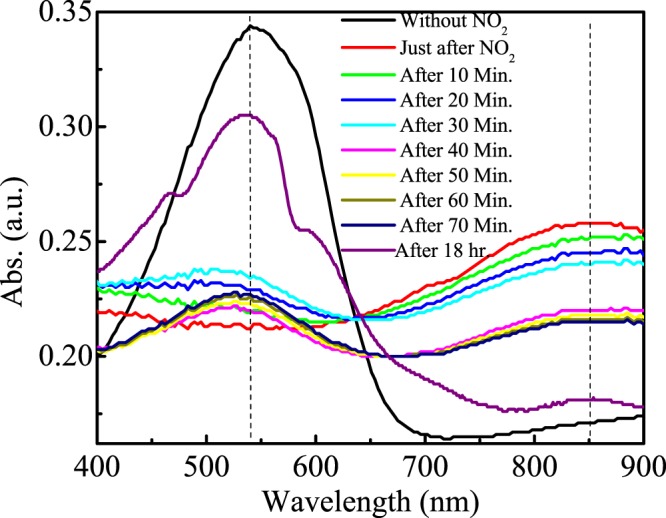
Electronic absorption spectra of unexposed and NO_2_ exposed hybrid PBTTT/GO nano-composite thin film as a function of time.

In order to probe the effect of NO_2_ exposure on PBTTT/GO nano-composite film as a function of time as demonstrated in Fig. 3, intensive electronic absorption spectra were recorded. It is important to mention here that widening and moving of red spectra of prepared films in comparison with its solution form are arisen due to the charge exchange (CE) phenomenon between the polymer chains and among the polymer chains and GO nanosheets. However, the level of intrachain ordering and increase in conjugation length result the enhancement of the proportion of lowest energy peak and next reproduction peak (A_0–0_/A_0–1_). In order to investigate the effect of NO_2_, we exposed our composites film in NO_2_ ambient for few minutes, and recorded spectrum with fixed interval of time. Absorption spectrum of just exposed films demonstrates visible differences as compare to unexposed film. It demonstrates the peak broadening with decrease in peak intensity of major peak appeared at 540 nm and appearance of new peak at 850 nm. Since, NO_2_ have tendency to withdraw the electron from polymer backbone and form a charge transfer complex. Therefore, this change in absorption spectrum can be attributed to the interaction of NO_2_ with films and formation of polymer/NO_2_ charge complexes. It was observed that 540 nm major peak is again appearing as time passes. We have conducted this spectrum measurements experiment upto 18 hrs. and found that 850 nm peaks almost disappear and 540 nm peaks recover upto a mark with appearance of two clear shoulder peaks as demonstrated in Fig. 3. This phenomenon further validates those NO_2_ forming unstable charge transfer complexes which validate our previous recovery results.

#### Structural characterization

**Figure 4:**
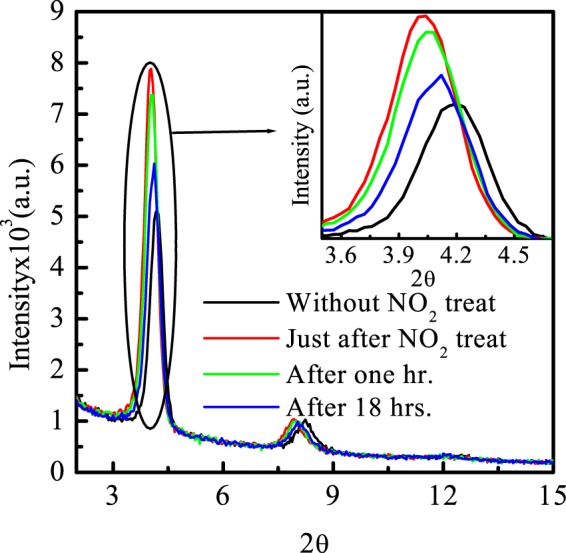
GIXD of unexposed and NO_2_ exposed PBTTT/GO nano composite film as function of time with grazing angle 0.2^0^. Inset shows the zoom image of (100) peak.

The GIXD pattern of as fabricated PBTTT/GO thin film has been recorded with 0.2° grazing angle as a function of time, which is shown in Fig. 4. The instrument is equipped with in plane diffractometer, while detector is allowed for out of plane movement. Here, the inset shows the zoom image of (100) peak as function of time. The obtained GIXD pattern of unexposed composite thin film shows the (100), (200), (300) diffraction peaks which are appeared at lower angle and having the single set of reflection (h00) is illustrated in Fig. 4. However, there is no signature of any GO peaks in composite film which validates the very less percentage (<3%) of GO content in polymer matrix. The appearance of similar peaks indexed at (100), (200), (300), with shifting towards lower angle and high intensity were observed due to just after exposure. The effect of exposure on film was studied for 18 hrs. and found to be gradually reaching towards its original position. The shifting of (h00) peak with enhanced intensity towards lower angle just after exposure reveals the increment in inter planner spacing as well as crystallinity as shown in Table 2. It is important to note that the observed changes is not stable with due course of time. It tries to regain its initial state as time passed as shown in inset of Fig. 4. It is quite obvious that increase in spacing is arising due to the doping of NO_2_ from alky chain side.

**Table 2:** Crystal parameters of unexposed, NO_2_ exposed and recovered samples.

Sample	Planes	FWHM	2 θ	d_100_ spacing
Pristine	100	0.384	4.21	21.64
	200	0.521	8.24	
Just after exposure	100	0.326	4.02	25.49
	200	0.440	7.92	
Recovery (After 18 hrs.)	100	0.366	4.06	22.70
	200	0.444	8.01	

#### Raman spectra

**Figure 5:**
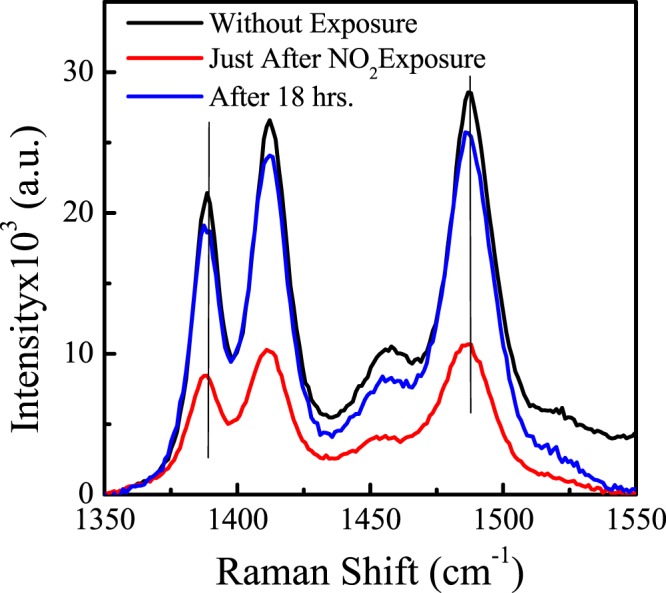
Raman spectra of PBTTT/GO nano composite thin film, just after NO_2_ exposure, and recovery after 18 hrs., respectively.

In order to probe the effect of NO_2_ on vibrational property of different bond appear in PBTTT, Raman measurements are carried out of the SS film as shown in Fig. 5. Moreover, Raman measurement was explored to examine effect of NO_2_ in three conditions such as pristine, just after exposure, and recovery (after 18 hrs). It is well reported in literature that peak position is directly governed by presence of bond in molecule. In Raman spectrum, the intensity of peak defines the polarizability of bonds while characterizing for the conjugated polymers. The orientations as well as degree of ordering/ crystallinity are specifically connected with the film preparing conditions and procedure incorporated. From Table 3 and Fig. 5, it is quite obvious that there is shifting of peak towards lower energy with reduction in peak intensity for just after exposure and trying to regain its original position with time with enhancement in peak intensity. Noh *et al*.^40^ have reported that attachment of foreign molecule with polymer backbone causes reduction in polarizability which results in decrease in peak intensity. Ram *et al*.^45^ have reported that NO_2_ have tendency to withdraw an electron to sulfur atom and create hole over polymer backbone, and act as dopant. This whole have tendency to delocalize over whole polymer chain up to the conjugation length. The delocalization of holes cause reduction in polarizability and reduction in peak intensity. Further, we reordered the Raman spectra of same sample after 18 hours which reveals the increasing of peak intensity with regain (not 100%) of peak position.

**Table 3:** Peak assignment using Raman Spectra.

Samples →Peak Assignment↓	Pristine	Just exposure	Recovery
C-S-C	740	736	736
Thiophene C-C	1389	1387	1388
Thienothiophene C=C stretch	1412	1412	1412
Interring C-C stretch	1458	1456	1458
Thiophene C=C stretch	1487	1486	1487

#### Electrochemical cyclic voltammetry

**Figure 6:**
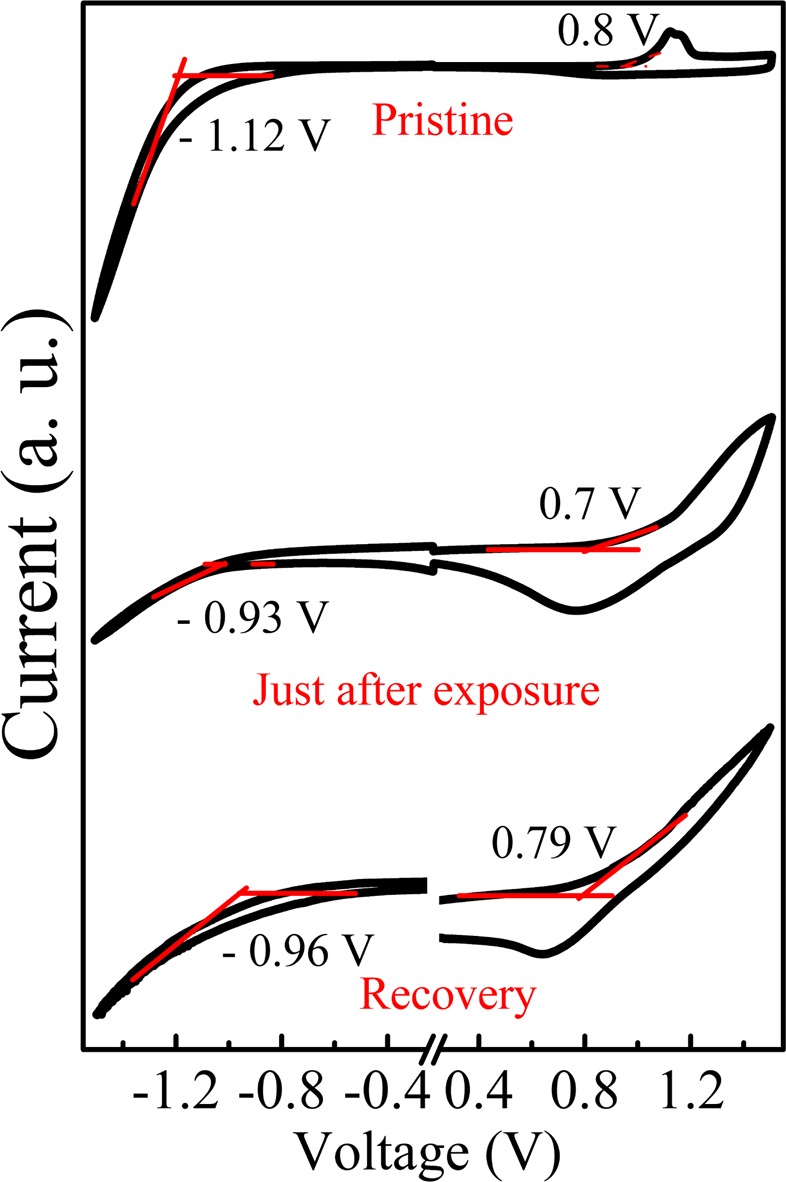
Cyclic voltammetry of unexposed, just after exposure and, recovered after 18 hrs.

The electronic property of fabricated hybrid thin film, such as determination of HOMO-LUMO level, energy band gap, and CV measurement has been recorded. Preferably, CV measurement is adapted to measure the oxidation potential for the estimation of the HOMO level of SS film as illustrated in Fig. 6. It could easily be understood that the band gap, LUMO, and HOMO (work function) of nano hybrid conjugated polymers are directly governed with thin film processing techniques being adapted. Table 4 shows the HOMO, LUMO and band gap for pristine; NO_2_ exposed and recovered PBTTT/GO nano composite film. The HOMO and LUMO level of as fabricated (pristine) film have been calculated using the equation E_HOMO_ = −e(4.41+E_ox(onset)_)V and measured to be −5.4 eV. Further, the LUMO level has been estimated using the formula E_LUMO_+Eg and measured to be −3.48 eV. It is quite obvious from Table 4 that adsorption of NO_2_ with nano hybrid film envisage the reduction in band gap as result of shifting of electronic energy level from 1.92 eV to 1.63 eV. This reduction in band gap has already been observed in electronic absorption spectra. Further, the adsorption of NO_2_ is not permanent with nanohybrid films and desorption of gas occurs with time which causes the recovery of nanohybrid film. It is noteworthy from electronic absorption spectra that our sample does not recover fully upto its original position. Therefore, we obtained the band gap of the film as 1.75 eV after 18 hrs. of recovery time.

**Table 4:** Electronic parameters extracted from CV.

Sample	E_HOMO_ (eV)	E_LUMO_ (eV)	E_gap_ (eV)
Pristine	−5.4	−3.48	1.92
Just after exposure	−5.3	−3.67	1.63
Recovery	−5.39	−3.64	1.75

#### Atomic force microscopy

**Figure 7:**
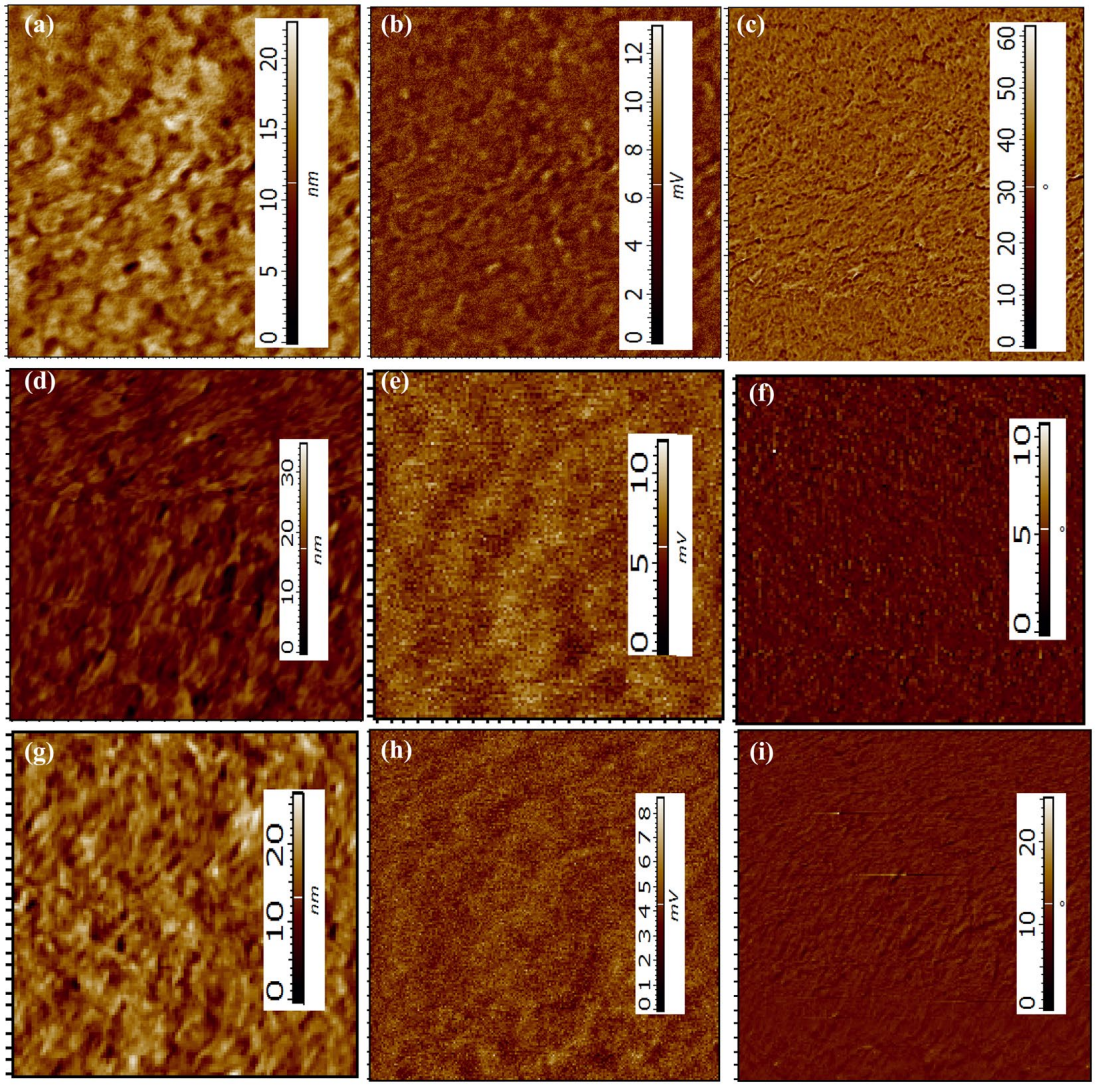
AFM topography, KPFM and phase image of **(a–c)** unexposed, **(d–f)** just after NO_2_ exposure, and **(g–i)** recovered nanohybrid composite film prepared via SS method.

In order to examine the effect of NO_2_ on surface morphology of PBTTT/GO nanohybrid film, we recorded the AFM height image, KPFM, phase image in different condition such as pristine, just after NO_2_ exposure and recovered samples after 18 hrs. as shown in Fig. 7. The inherent structural and morphological properties acquired by the thin film grown via SS method offers better gas adsorption and desorption that can be directly correlated with the sensor performance. The AFM images for before and after exposure of NO_2_ is illustrated in Fig. 7, indicating the effect of oxidizing gas on the film morphology (Scan area 5 μm × 5 μm). It is important to mention here that AFM height, KPFM and phase image were recorded contemporarily. AFM height image recorded in three conditions demonstrate apparent morphological differences with a bigger domain size and height for just exposed film. It is apparent from image that pristine and recovered films have almost similar height. RMS surface roughness analysis demonstrates 2.7 nm, 3.0 nm and 2.75 nm respectively for pristine, just exposed and recovered samples which is very much similar to height image. Further, KPFM was recorded in the same scan area for comparing the surface potential contrast (SPC) of the targeted film surface. Figure 7(c,f) clearly endowed that the phase morphology of the targeted film is not similar in entire cases. The measured height of SPC morphology of the unexposed composite film obtained from KPFM reveals small domains. However, exposure causes change in SPC morphology. Surface potential contrast analysis demonstrates 0.85 mV, 1.31 mV and 0.91 mV rms surface roughness respectively for pristine, just exposed and recovered samples. These variations in the SPC morphology between the films arise only due to change in electronic property after adsorption of NO_2_. This collectively summarises the differences observed in the electronic property as well as surface potential of the films that could further be confirmed by CV measurement. KPFM images of same region demonstrate the clear difference in potential distribution of surface. The surface properties of the films were also examined through phase imaging by monitoring the change in phase angle and found the visible difference. These morphological difference further validates our previous results obtain via multiple characterization techniques.

## Discussion

### Mechanism of NO_2_ sensing in PBTTT/GO nanohybrid

**Figure 8:**
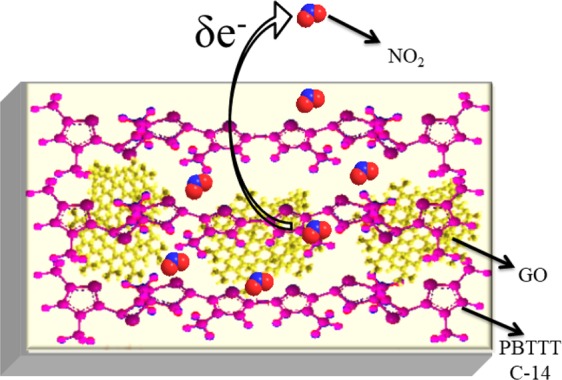
Sensing mechanism and recovery of nano composite film.

In order to understand such a swift response with high sensitivity, we propose a possible mechanism as illustrated in Fig. 8 which demonstrate the PBTTT polymer chain and GO nanosheets. The charge trapping and doping phenomenon are broadly described as sensing mechanism to explain the sensor response towards NO_2_ gas^2^. Since the conductivity of PBTTT based nano composite film is getting changed after exposure to NO_2_, which may be possible due to “doping effect” of nano composite film by oxidizing it. The proposed sensing mechanism with exposure of NO_2_ gas is shown in Fig. 8. This includes the process of nano composite film -NO_2_ interaction on the top of the surface of the film having high surface-to-volume ratio. When the NO_2_ molecules interact with the nano composite film, the NO_2_ (acts as an electron-acceptor) gas withdraw electrons from the top of the HOMO (Highest occupied molecular orbit) level of PBTTT and GO nanosheets. It forms NO_2_^−^ ions and positive charges (holes) which is delocalised over whole polymer chains and sheets. The interactions of NO_2_ molecule with PBTTT semiconducting polymer have been described schematically in Fig. S5. The populated mobile charge carriers (i.e. holes) have been observed across the GO nanosheet and backbone of the polymer, due to the creation of additional energy levels in between the HOMO and the LUMO (Lowest occupied molecular orbit) level. This results the reduction in band gap, which is further validated by absorption spectrum. Further, 850 nm peak in UV-vis. arises due to charge transfer interaction between polymer backbone, and NO_2_ molecule; which further confirm the molecular doping in polymer matrix. Hence, the conductivity of PBTTT/GO film would increase. It is important to mention here again that concentration of GO is very much low (<3%) in polymer matrix as confirmed by GIXD. The sensor shows swift response and fast recovery of target gas which may be possible due to the speedy adsorption process incurred over the surface of nanohybrid thin film. In contrary, the process of diffusion phenomenon is happened on the bulk films. Here, the optimized thickness of composite thin film results the adsorption phenomenon which is dominating over diffusion. The increasing separation of (100) planes also validates the adsorption of NO_2_ between the alkyl side chains.

## Conclusion

We have successfully explored the facile SS method for fabrication of large area, highly oriented active matrix of PTFTs using PBTTT/GO nano composite-hybrid. The sensor shows practical real time sensing application with highly selective as comparable with the metal oxide sensors counterparts at low concentrations also. It has been observed that % change in drain current (sensor response) saturate with increasing concentration of NO_2_. Our sensor also demonstrates pretty good stability, response and recovery time (1.2 and 9.5 minutes for 10 ppm). A detailed insitu investigation of morphological, spectroscopic, electronic properties of the nanohybrid composite films, envisaged the interactions of NO_2_ molecules with polymer/GO matrix, which was less studied in the past. These multiple characterization demonstrates the significant spectral, morphological, structural changes after adsorptions of NO_2._ The swift response and fast recovery of target gas may be possible due to the speedy adsorption process incurred over the surface of nanohybrid thin film. Further, we have proposed a sensing mechanism and exact site of adsorption of NO_2_ at composites film. The endeavour made on this work opens up the path to further enhancements in the sensors performance with the use of suitable hybrid polymer/2D-materials nanocomposite thin film as comparable with nanostructured metal oxide and their composite based sensors. Thus, our study has a pave a path for fabrication of real time room temperature operated gas sensor and give an insight about the sensing mechanism, and exact adsorption site of NO_2_. On the basis of obtained results, one can develop a polymer nanocomposite based low cost, high performing NO_2_ sensors at room temperature in near future.

## Methods

### Synthesis of GO, GO/PBTTT nanohybrid solution and fabrication of nanohybrid thin film via SS method

The chemical used for conjugated polymer PBTTT C-14, having molecular weight (>40,000) was directly purchased from Sigma-Aldrich and used without any further adultration. GO was chemically synthesized by improved Hummer’s method and characterized for confirmation of formation of GO (Supporting Information). The biphase transferred methodology was adopted to transfer GO from de-ionised water to chloroform (CHCl_3_) for the formation of 10 mg/ml PBTTT/GO nano hybrid solution where 1 mg/ml stable dispersions of nanosheets was prepared in water. Further, the same concentration by volume of CHCl_3_ was added which results clear bi-phase where CHCl_3_ remain in bottom due to its heavy weight. In order to transfer the GO from water, it was sonicated for 1 hr. This chlorofom with uniformly dispersed GO was further used for formation 10 mg/ml PBTTT/GO nanohybrid solution. A 10 μl of nanohybrid solution was dropped over high surface tension mobile liquid substrate. Due to volatile nature of CHCl_3_ and opposite nature of liquid substrate and solution, CHCl_3_ gets spreads spontaneously over the liquid substrate due to surface tension gradient and solidify rapidly after quick evaporation of CHCl_3_ and solid film remain over the liquid substrate. The details of nucleation and growth phenomena of nanohybrid film over liquid substrate have been discussed in Results and discussion section. The thickness of the film as grown over the solid substrate was measured via AFM tip and found in the range of 15 ± 1 nm in each case.

### Characterization tools and sensing measurement

**Figure 9:**
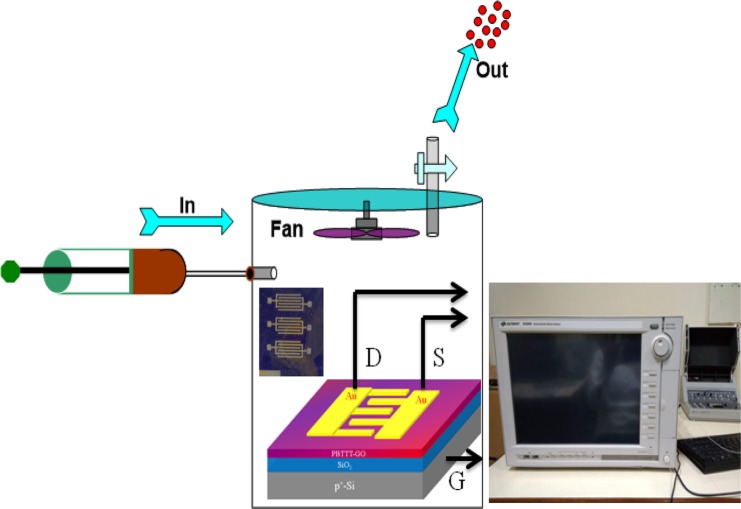
Schematic representation of gas sensing set-up. Zoom in image of fabricated sensor shows the real image of PTFT which has been kept inside the sensing chamber used for NO_2_ detection.

In order to interrogate the effect of NO_2_ exposure on the composite polymer thin film, an interdigitated Au metal electrodes act as source-drain structure (Au/PBTTT-GO nanohybrid/Au) over insulating gate (SiO_2_) with channel length 200 μm, and width 31.6 mm were created by physical vapour deposition i.e. vacuum evaporation coating system (Hind HIVAC” model No-12A4D, India) through shadow masking as shown in Fig. 9. The charge transfer characteristics of fabricated sensors under different concentration of NO_2_ were investigated using Keysight parameter analyser (B1500A). The common source configuration of PTFTs has been deployed to analyse the sensor’s performance. The surrounding environment has been kept at room temperature 25 °C and relative humidity (RH) of 54% during the sensing measurements. The schematics of whole measurement set up have been illustrated in Fig. 9. An *in situ* sensing measurement was carried out in this indigenously developed closed chamber. Further, in order to understand the sensing mechanism, we conducted the *in situ* spectral, structural, electronic and morphological investigation of nanohybrid film. The electronic absorption spectra were recorded using Biotech single beam spectrophotometer (model epoch- 2, USA). The Raman spectroscopy was also recorded using a μ Raman Spectrometer (Renishaw, Germany) with a 532 nm line of laser at 1 mW. It has been shown that Raman characteristics undergo very distinct changes in the case of pristine, exposed and recovery and also supported by FTIR spectra. For structural analysis of nanohybrid film, an advanced thin-film X-ray diffraction system (also known as GIXD (Rigaku, Japan)) is used. This instrument is equipped with grazing incidence in-plane diffraction with focused Cu Kα radiation (λ = 1.54056 Å). Each sample was mounted with an angle of incidence at 0.2^0^ to record the phases available in the film scanning area. The AFM topographies of PBTTT/GO nano hybrid films fabricated by SS technique over ITO substrate were probed under tapping mode of the instrument (make NT-MDT, Russia).

## Supplementary information

Supplementary Information.
