# Supplementary material for: Polymer/Graphene oxide nanocomposite thin film for NO2 sensor: An in situ investigation of electronic, morphological, structural, and spectroscopic properties
Source: Sci Rep. 2020 Feb 19;10:2981. doi: 10.1038/s41598-020-59726-5 (PMC7031537; doi:10.1038/s41598-020-59726-5)
Supplement: Supplementary file 1 — Supplementary Information. [file 41598_2020_59726_MOESM1_ESM.doc]

**Electronic Supplementary Information**

**Polymer/Graphene oxide nanocomposite thin film for NO_2_ sensor: An in situ investigation of electronic, morphological, structural, and spectroscopic properties.**

Praveen Kumar Sahu^a^, Rajiv Kumar Pandey^b^*, R. Dwivedi^a^, V. N. Mishra^a^, and R. Prakash^b^*

^a^Department of Electronics Engineering, Indian Institute of Technology (Banaras Hindu University), Varanasi-221005, India

^b^School of Materials Science and Technology, Indian Institute of Technology (Banaras Hindu University), Varanasi-221005, India

Corresponding Author: - [pandeyrajiv05@gmail.com](mailto:pandeyrajiv05@gmail.com) (R. K. Pandey), [rprakash.mst@iitbhu.ac.in](mailto:rprakash.mst@iitbhu.ac.in) (R. Prakash).

1. **Characterization of GO**


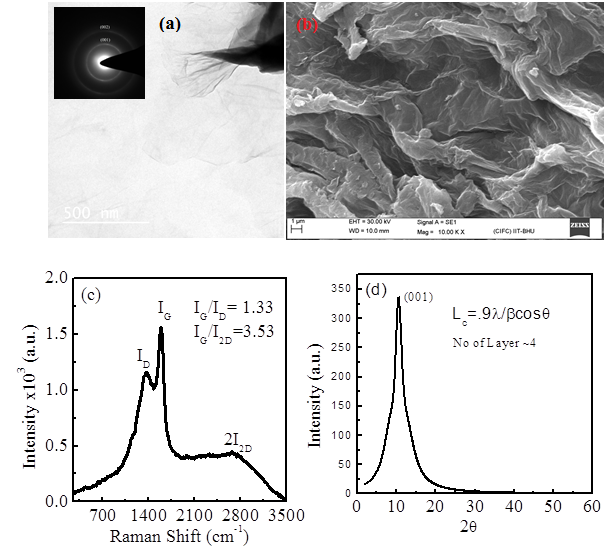


**Figure S1.** (a) TEM image of GO. (Inset shows the SAED pattern), (b) FESEM image of GO, (c) Raman spectrum and (d) XRD of GO.

1. **Characterization of PBTTT/GO Nanocomposite film**

**
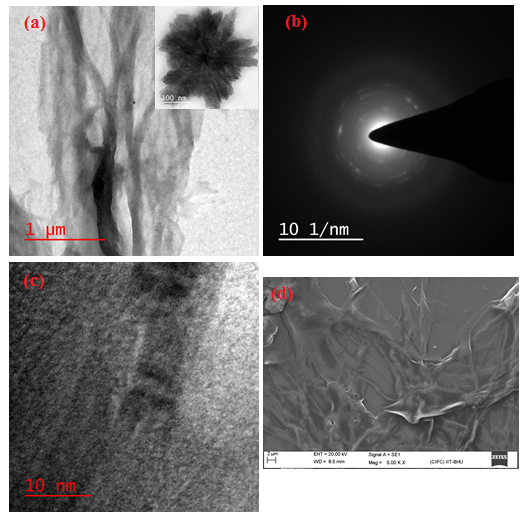
**

**Figure S2.** (a) TEM image of PBTTT/GO nanocomposite. (Inset shows the higher magnification TEM image of same), (b) SAED pattern of same film, (c) HRTEM image of PBTTT/GO Nano composite, and (d) SEM image of PBTTT/GO nanocomposite film.

1. **Electrical characterization of PBTTT/GO nanocomposite film**


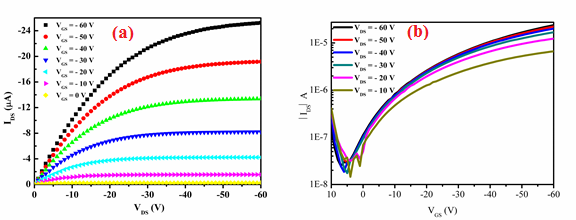


**Figure S3.** (a) I_d_-V_d_ curve, (b) I_d_-V_g_ curve before NO_2_ exposure.

1. **Effect of increasing concentration of NO2 on sensor :**

**
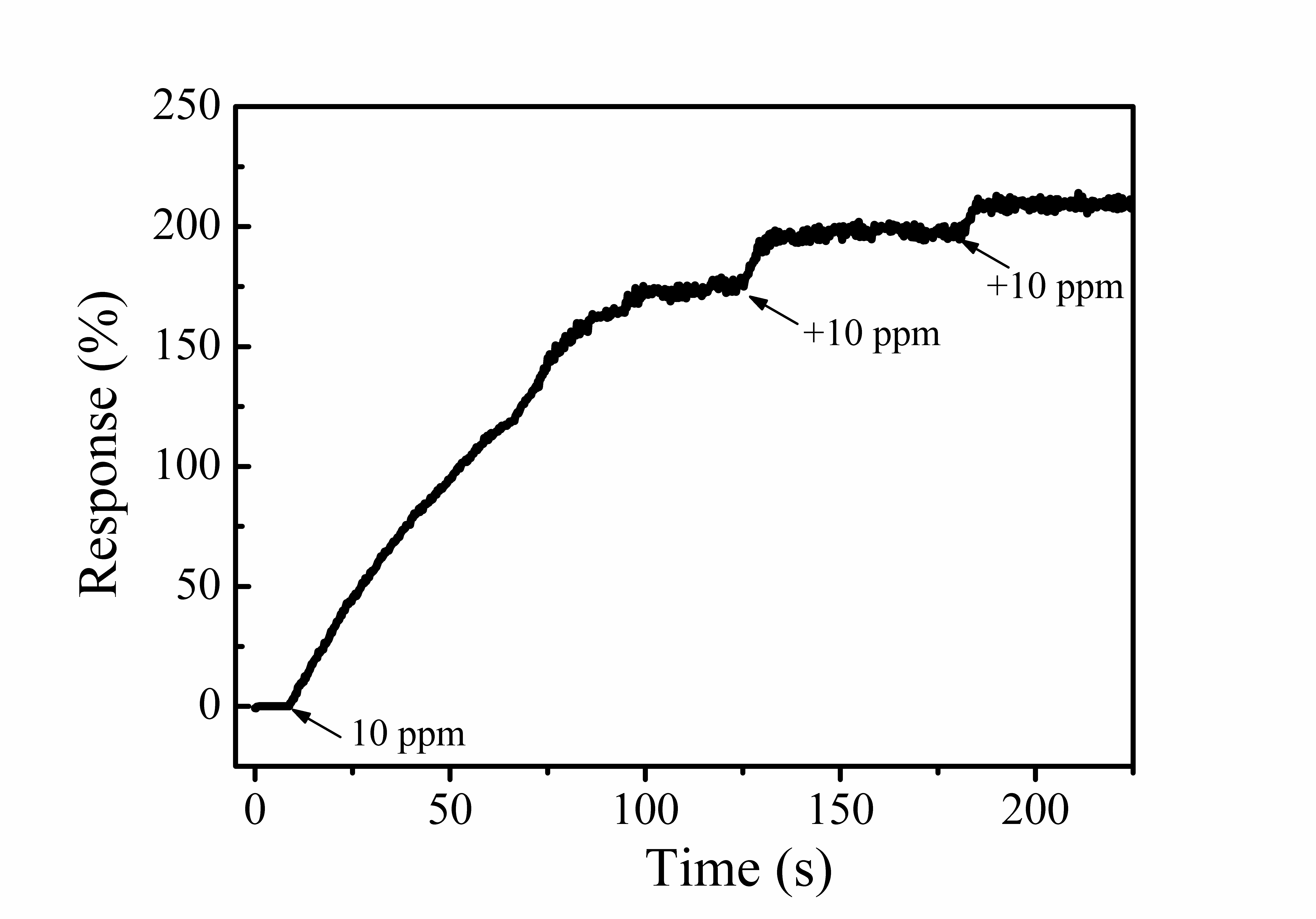
**

Figure S4. Effect of increasing concentration of NO_2_ gas on as fabricated PTFTs sensor.

1. **Sensing mechanism of as fabricated PTFT sensor:**

**
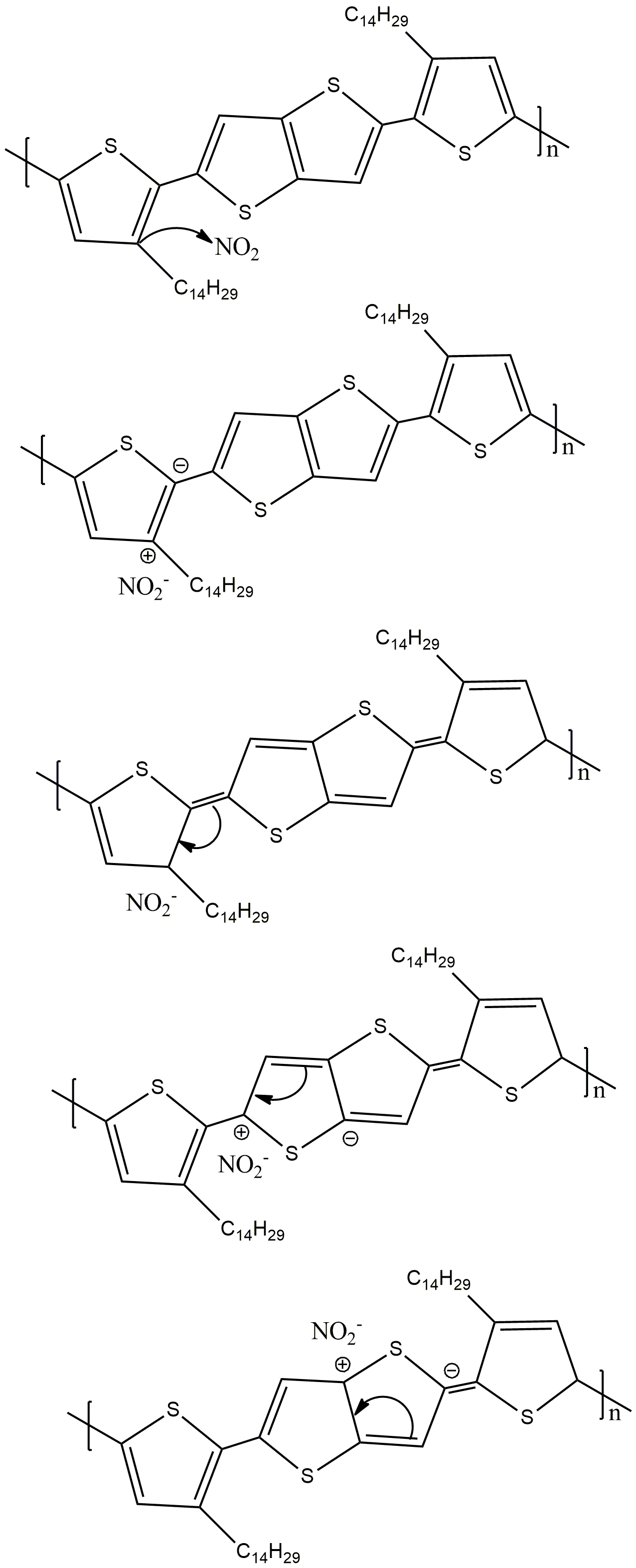
**

**
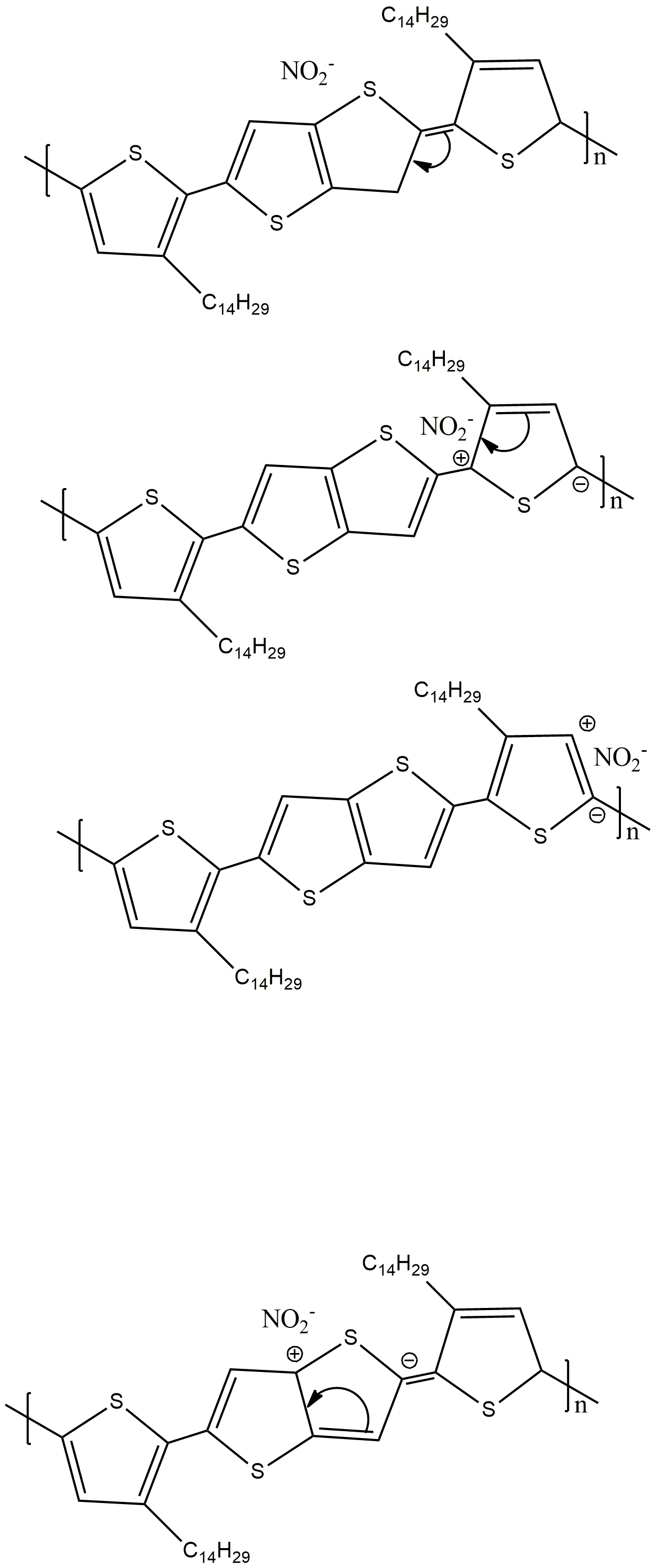
**

**Figure S5.** The process of interacting the NO2 molecule with PBTTT polymer: electrons withdrawing from the PBTTT to NO2 molecules→ the formation of NO_2_ - ions and positive charges (holes), → the establishment of new double bond, → the transfer procedure of holes created by the NO_2_ molecule.

1. **FTIR Spectra**

As shown in Fig. S6, the FTIR spectra of PBTTT/GO nanocomposite can be observed at 769 cm^−1^ [C-H out of plane deformation], 945 cm^−1^ [unsaturated C-H vibration], 1144 cm^−1^ [the C–H in plane deformation], and 1513 cm^−1^ - 1653 cm^-1^ [associated to C = C]. The antisymmetric and symmetric C = C stretching vibration mode of thiophene ring was observed at 1555 cm^-1^ and 1653 cm^-1^ respectively. The wavenumbers, 1241 cm^-1^ is usually attributed to the C-OH stretching vibrations, 1016 cm-1 is assigned to C-O (epoxy) groups and 1700 cm^−1^ corresponds to the carbonyl stretching of GO sheet. Apparently, the spectra of pristine PBTTT/GO nanocomposite seems to be similar to that of recovered sample after exposing with NO2 gas within 18 Hrs. of exposure, which indicate that the properties of nanohybrid PBTTT/GO film approaching for recovery.

**
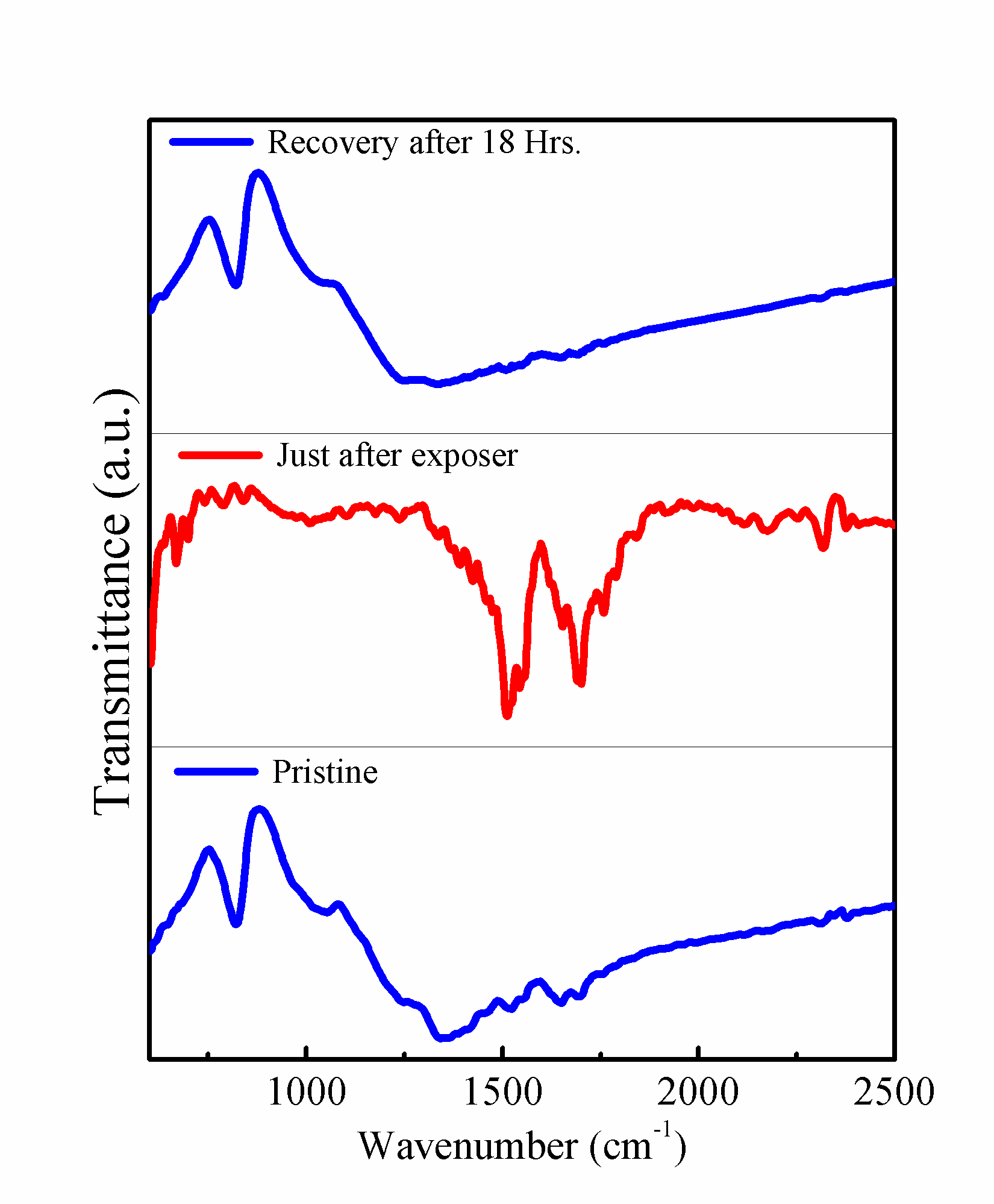
**

**Figure S6.** FTIR spectra of PBTTT/GO nanocomposite: (a) Pristine, (b) just after exposure and (c) recovery after 18 Hrs. of exposure**.**
